# Vector-Borne Pathogens with Veterinary and Public Health Significance in *Melophagus ovinus* (Sheep Ked) from the Qinghai-Tibet Plateau

**DOI:** 10.3390/pathogens10020249

**Published:** 2021-02-22

**Authors:** Qing-Xun Zhang, Ye Wang, Ying Li, Shu-Yi Han, Bo Wang, Guo-Hui Yuan, Pei-Yang Zhang, Zi-Wen Yang, Shuang-Ling Wang, Ji-Yong Chen, Hai-Shun Zhong, Xue-Qing Han, Hong-Xuan He

**Affiliations:** 1 National Research Center for Wildlife-Born Diseases, Institute of Zoology, Chinese Academy of Sciences, Beijing 100101, China; zhangqingxun@ioz.ac.cn (Q.-X.Z.); yew4315@126.com (Y.W.); hanshuyi@ioz.ac.cn (S.-Y.H.); wangbo@ioz.ac.cn (B.W.); yuanguohui1022@163.com (G.-H.Y.); zhangpeiyang@ioz.ac.cn (P.-Y.Z.); yangziwen@ioz.ac.cn (Z.-W.Y.); wangsl0830@163.com (S.-L.W.); 2 National Research Center for Wildlife-Born Diseases, University of Chinese Academy of Sciences, Beijing 100049, China; 3 Ningxia University, Yinchuan 750021, China; 4 State Key Laboratory of Plateau Ecology and Agriculture, Qinghai University, Xining 810016, China; yingli@126.com; 5 Animal Disease Prevention and Control Center of Yushu, Yushu 815099, China; 13997361966@163.com; 6 Animal Husbandry and Veterinary Station of Xunhua, Xunhua 811100, China; zhs9822@163.com; 7 Chinese Academy of Inspection and Quarantine, Beijing 100176, China; xqhancaiq@163.com

**Keywords:** *Melophagus ovinus*, vector-borne pathogens, occurrence, reservoir, China

## Abstract

*Melophagus ovinus* (sheep ked) is a hematophagous ectoparasite that mainly parasitizes sheep. In addition to causing inflammation, wool loss, and skin damage to the animal hosts, *M. ovinus* also serves as a vector for a variety of pathogens and is highly likely to participate in the life and transmission cycle of pathogenic organisms. Herein, we investigated the presence and molecular characterization of vector-borne pathogens in *M. ovinus* from Qinghai-Tibet Plateau, China. A total of 92 *M. ovinus* pools collected from the Qinghai province of China were screened for the presence of selected vector-borne pathogens. The overall positive rate of *A. ovis*, *A. bovis*, *A. phagocytophilum*, and *T. ovis* in *M. ovinus* was 39.1%, 17.4%, 9.8%, and 89.1%, respectively. All of the samples were negative for Border disease virus (BDV), other *Anaplasma* species, *Babesia* spp., *Rickettsia* spp., and *Borrelia* spp. Co-infection of different *Anaplasma* species and *T. ovis* occurred in 51.2% of all samples with *T. ovis*. The positive rates of *A. ovis*, *A. bovis*, and *A. phagocytophilum* in different regions and altitudes of the sampling sites were significantly different. Sequence and phylogenetic analysis of target genes confirmed their identity with corresponding pathogens. Our results elucidate the occurrence and molecular characterization of *Anaplasma* spp. and *Theileria* spp. in *M. ovinus*, which could act as potential zoonotic reservoirs. To the best of our knowledge, this is the first report of the detection of *A. bovis* and *A. phagocytophilum* DNA in *M. ovinus*. This study gives the first extensive molecular survey of vector-borne pathogens with veterinary and public health significance in *M. ovinus* from the Qinghai-Tibet Plateau, China.

## 1. Introduction

*Melophagus ovinus* (sheep ked) belongs to the family Hippoboscidae (Diptera: Hippoboscoidea) and is a blood-feeding ectoparasite of livestock and wild animals, including sheep, goats, rabbits, dogs, Tibetan antelope, European bison, and red foxes, and has also been found in humans [1,2,3]. The life cycle of sheep ked comprises the larva, pupa, and wingless adult stages, and all life stages of this ectoparasite occur on the host. After mating, the females produced single larvae every 6–8 days that were attached to the host wool and molded into the puparial stage within 6–12 h. It takes the pupae 19–30 days to develop into an adult [3]. Adults are commonly circulated among the animals and transferred from ewes to their offspring by direct contact. *Melophagus ovinus* (*M. ovinus*) is reported to cause inflammation, wool loss, skin damage, and reductions in weight gain of sheep and has significant economic effects in the sheep industry [4,5]. Of the sheep studied, 61–81% of sheep were infested with *M. ovinus* [3]. *Melophagus ovinus* is broadly distributed in Africa, Europe, Oceania, North America, and Asia [5]. In China, *M. ovinus* has recently been reported to parasitize sheep and Tibetan antelopes in Tibet, Xinjiang, Qinghai, and Gansu, and was also detected in imported sheep and sheep wool in certain areas of China [6,7,8].

*Melophagus ovinus* serves as potential vectors of a variety of pathogens and has been reported to be responsible for the transmission of pathogenic organisms such as helminths, protozoa, bacteria, and viruses due to their blood-feeding behavior towards hosts [1,9]. *M. ovinus* was reported to mechanically transmit the Bluetongue virus in sheep [2]. Additionally, previous studies showed that *M. ovinus* may be a vector for *Bartonella schoenbuchensis* and *B. chomeli* in the USA [10], *Anaplasma ovis* in Hungary [11], *Acinetobacter* spp. in Ethiopia [12], and *Bartonella* in Central Europe [2]. Chu et al. [6] reported *Borrelia burgdorferi* sensu lato in sheep keds in Tibet, China. Recently, in China, *Anaplasma ovis* [13], *Rickettsia raoultii* and *R. slovaca* [5], *Theileria ovis* [9], and Border disease virus (BDV) [14] have also been detected in *M. ovinus* in the Xinjiang Uygur Autonomous Region of northwestern China.

The Qinghai province is one of the traditional animal husbandry bases in China and a small number of reports have recorded the presence of *M. ovinus* in the region [5]. However, very little is known about the occurrence of arthropod-borne pathogens in *M. ovinus* from the Qinghai. Given the veterinary and public health significance of *M. ovinus*, the objective of the present study was to investigate the presence of vector-borne pathogens in *M. ovinus* from the Qinghai-Tibet Plateau of China.

## 2. Results

**Figure 1:**
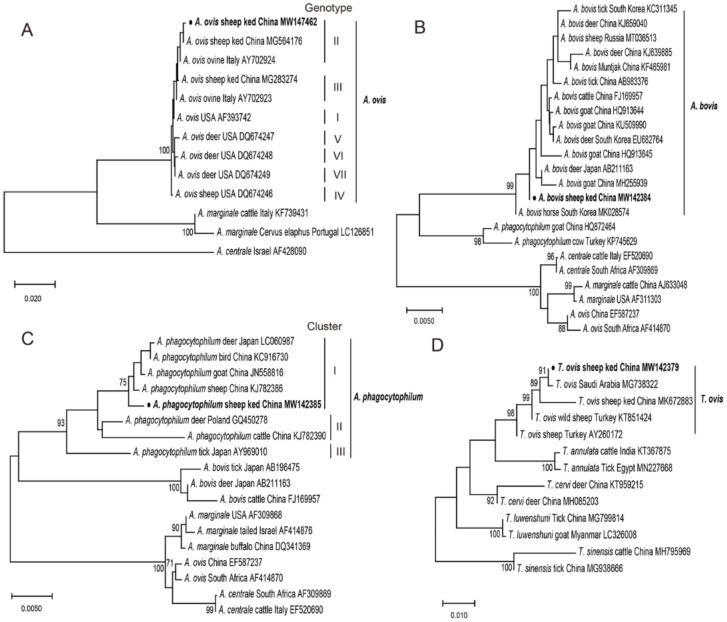
Phylogenetic relationship of partial segment msp4 of *A. ovis* (**A**), 16S rRNA gene for *A. bovis* (**B**), *A. phagocytophilum* (**C**), and 18S rRNA gene of *T. ovis* (**D**) identified in the present study and reference strains. All molecular phylogenetic trees were constructed by the neighbor-joining method with Kimura 2-parameter model using the MEGAX software, and the bootstrap test was assessed with 1000 replicates. The species identified in this study are indicated by ● and highlighted in bold.

A total of 92 *M. ovinus* pools were screened for the presence of selected vector-borne pathogens. Of the 92 samples tested, 46 (50.0%) pools were positive for one or more *Anaplasma* species. The infection rates were 39.1%, 17.4%, and 9.8% for *Anaplasma ovis* (*A. ovis*), *Anaplasma bovis* (*A. bovis*), and *Anaplasma phagocytophilum* (*A. phagocytophilum*) in *M. ovinus*, respectively (Table 1). Importantly, *A. bovis*, and *A. phagocytophilum* were detected in *M. ovinus* for the first time. A total of 82 pools (89.1%) were positive for piroplasm infections, and all of which belonged to *Theileria ovis* (*T. ovis*). No positive results were obtained for other tested pathogens, including BDV, *Anaplasma centrale*, *Anaplasma platys*, *Anaplasma capra*, *Anaplasma marginale*, *Babesia* spp., *Rickettsia* spp., and *Borrelia* spp. Mixed infections of both the *T. ovis* and *Anaplasma* species accounted for 51.2% (42/82) of all samples with the *T. ovis. Anaplasma ovis* co-infections with *A. bovis* and *A. phagocytophilum* accounted for 26.1% (12/46) and 2.2% (1/46) of *Anaplasma* species infections, respectively. Sequence analysis of the msp4 sequences of *A. ovis* (sequence similarity 100%), 16S rRNA sequences of *A. bovis* (sequence similarity 99.9–100%) and *A. phagocytophilum* (sequence similarity 100%), and 18S rRNA gene sequences of *T. ovis* (sequence similarity 99.9–100%) confirmed their identity with corresponding pathogens by using BLASTn search. Phylogenetic analysis of the msp4 sequences represented showed that the MW147462 sequence was classified as *A. ovis* Genotypes II based on nucleotide mutation sites (A^360^T^366^G^400^). Sequence MW142385 of *A. phagocytophilum* was classified into cluster I, and sequence MW142384 of *A. bovis* was identical with strains isolated from sheep (MT036513), tick (KC311345), horse (MK028574), and deer (KJ659040) (Figure 1A–C). The phylogenetic analysis of the 18S rRNA gene confirmed that the detected piroplasm (MW142379) was *T. ovis* (Figure 1D).

Risk factors including *M. ovinus* gender, the region, and altitude of the sampling sites were used as variables for statistical analysis of the infection patterns of *Anaplasma* spp. and *Theileria* spp. occurrence (Table 2). For the occurrence of *A. ovis*, *A. bovis*, and *A. phagocytophilum*, significant differences between locations have been observed. *Melophagus ovinus* collected in Haidong had a higher risk than other *M. ovinus* in Golog and Yushu to be infected with *A. ovis* (*p* = 0.01) and *A. bovis* (*p* = 0.003), while *A. phagocytophilum* infection rate in *M. ovinus* collected in Yushu was significantly higher than Haidong and Golog (*p* = 0.011). *Melophagus ovinus* collected at 3000 m areas was at higher risk of being infected with *A. ovis* (*p* = 0.033) and *A. bovis* (*p* = 0.007) than in *M. ovinus* collected at altitudes of 3800 m and 4100 m. Besides, the results showed no significant difference in gender.

## 3. Discussion

To date, few publications have described the distribution and prevalence of vector-borne pathogens in *M. ovinus* from the Qinghai-Tibet Plateau, China [5,6]. As the traditional animal husbandry base, epidemiological investigations into vector-borne pathogens with veterinary and public health significance in Qinghai are of particular importance. *Anaplasma* spp. occurrence in *M. ovinus* demonstrated a wide distribution of *A. ovis*, *A. bovis*, and *A. phagocytophilum* in the Qinghai-Tibet Plateau, China. *A. ovis* has been considered as the etiological agents of anaplasmosis of domestic ruminants and it has been widely detected in sheep, goats, wild deer, and many tick species around the world [15,16]. In previous reports, all sheep keds (100%, 81/81) were found to harbor *A. ovis* in Hungary [11] and 28 specimens (including five pupal specimens) (31.8%, 28/88) collected in 2016 and 2017 in Xinjiang, China tested positive for *A. ovis* [13]. The positive rate (39.1%) and genetic characteristic (Genotype I) of *A. ovis* in *M. ovinus* in this study concurred with other reports published in Xinjiang.

To the best of our knowledge, this is the first molecular evidence of *A. bovis* and *A. phagocytophilum* in *M. ovinus* over the world. *Anaplasma bovis* mainly affects cattle with fever, progressive anemia, and even death, and the subclinical infections of this agent have also been found in small mammals and ruminants, indicating the reservoir competence of those animals for *A. bovis* [17]. Besides, *A. bovis* can be found in many tick species (*Haemaphysalis longicornis*, *Haemaphysalis lagrangei*, *Haemaphysalis concinna*, and *Rhipicephalus evertsi*, etc.) in Asia, Europe, and Africa [16,18]. We detected *A. bovis* with a positive rate of 17.4% in *M. ovinus* for the first time, which indicated that *M. ovinus* may be the potential reservoirs or maintenance hosts of this agent. Among the *Anaplasma* species detected, *A. phagocytophilum* is an emerging zoonotic pathogen of human and animal granulocytic anaplasmosis and can be transmitted to a wide range of mammals including humans, ruminants, horses, cats, dogs, rodents, birds, and reptiles through the bite of ticks [19]. In the present study, this is the first time that *A. phagocytophilum* DNA has been detected in *M. ovinus* using the molecular biological method. Statistical analysis indicated that *Aanaplasma* spp. infections showed significant correlations with the region and altitude of the sampling sites and the co-infection. Our results expand the potential vector spectrum of *A. bovis* and *A. phagocytophilum* and emphasize the veterinary and public health significance of *M. ovinus*.

Parasitic protists of the genus *Theileria*, especially *T. annulata*, *T. sergenti*, and *T. hirci*, are the causative agent of Theileriosis and have a wide geographical and host-species distribution. Among the *Theileria* species, *Theileria ovis* mainly causes benign theileriosis in sheep and goats, which is easily overlooked [20]. *Theileria ovis* are distributed widely in Asia, Europe, and Africa. In China, *T. ovis* has mainly been reported in animal, tick, and sheep keds from Xinjiang [9,21], Inner Mongolia [22], Qinghai [23], Sichuan [24]. Several reports have recently shown that there are two species of *Theileria* spp. (*T. ovis* and *T. luwenshuni*) in *M. ovinus* [9,24]. Historically, hard ticks were considered as the only and essential vector for *Theileria* spp. Our findings and similar studies conducted by Zhao [9] expand the potential vector spectrum of *T. ovis*. Herein, a high positive rate (89.1%, 82/92) of *T. ovis* DNA was demonstrated in *M. ovinus* in the present study, but this needs to be confirmed through more testing. Many factors including biogeography, the season of sample collection, number of samples, etc., may contribute to the differences between investigations of pathogenic organisms in *M. ovinus* in other regions in China or other countries.

Vector-borne pathogens including *Anaplasma* species, BDV, *Babesia* spp., *Rickettsia* spp., and *Borrelia* spp. cause economic losses in the livestock industry and pose a risk to humans. Although these infectious agents were negative in this study, some of these pathogens were found in tick, yak, and Tibetan sheep samples (unpublished data), implying that this region tends to have a higher risk of vector-borne diseases. Future studies should systematically screen for the presence of potential animal as well as human pathogens in *M. ovinus*.

We demonstrated the occurrence of *A. ovis*, *A. bovis*, and *A. phagocytophilum*, and *T. ovis* with veterinary and medical significance in *M. ovinus* in Qinghai, China. *A. bovis* and *A. phagocytophilum* was found for the first time and the present study extended the spectrum of pathogens potentially present in *M. ovinus*. The occurrence of these pathogens in *M. ovinus* may be a threat to animal and public health in the Qinghai-Tibet Plateau, China. Future investigations are warranted to elucidate the genetic diversity of vector-borne pathogens in *M. ovinus* and the role of *M. ovinus* as the specific biological vectors of some pathogens.

## 4. Materials and Methods

### 4.1. Study Sites and Sample Collection

**Figure 2:**
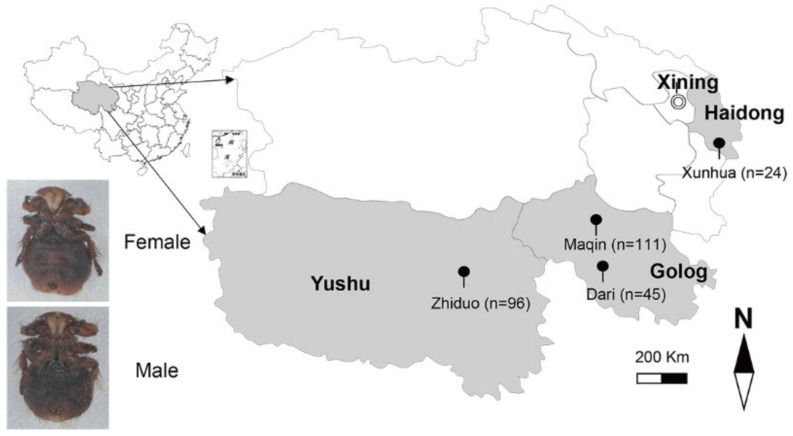
Sampling locations of *M. ovinus* (●) for the present survey in the Qinghai Province of China.

Adult sheep keds (n = 276) were collected at four sites: Xunhua, Haidong (n = 24, altitude 3000 m, 35°39′ N 102°41′ E), Maqin, Golog (n = 24, altitude 3800 m, 35°2′ N 99°12′ E), Dari, Golog (n = 24, altitude 4100 m, 33°43′ N 99°38′ E), and Zhiduo, Yushu (n = 24, altitude 4100 m, 33°37′ N 95°58′ E) during June 2020 in Qinghai province, China (Figure 2). After collection, sheep keds were shipped into the laboratory in cooled flasks and pooled (n = 92, three adults of the same sex collected from the same sheep were pooled) before being frozen at −80 °C until testing. Morphological studies (Figure 2) and 18S rRNA gene sequence analysis (data not shown) confirmed that the collected samples belong to sheep keds. The study was conducted in compliance with the ethical policies of the journal and the rules of the ethics committee of the Institute of Zoology, Chinese Academy of Sciences.

### 4.2. Nucleic Acid Extraction and PCR Amplification

All samples were sterilized with 70% ethanol and distilled water and were mechanically disrupted in 200 μL of PBS. Genomic DNA and RNA were extracted from 100 μL of the homogenate with the commercially TIANamp Genomic DNA Kit (TIANGEN BIOTECH (BEIJING) CO., LTD, Beijing, China) and Trizol reagent (Invitrogen, Carlsbad, CA, USA) according to the manufacturer’s protocol. cDNA was synthesized using the GoScript Reverse Transcription System and 5′-UTR of BDV was amplified according to the Access RT-PCR System (Promega, Madison, WI, USA) [14]. Nested PCR-based amplification was employed for the detection of *A. bovis*, *A. phagocytophilum*, *A. centrale*, *A. platys*, and *A. capra* based on the 16S rRNA gene and the citrate synthase (gltA) gene, and conventional PCR was used for detection of *A. ovis* and *A. marginale* based on the msp4 gene, as previously described [16,25,26,27]. For piroplasm (*Theileria* spp. and *Babesia* spp.) detection, all samples were screened using nested PCR assays targeting the 18S rRNA gene [27]. Other vector-borne bacteria including *Rickettsia* spp. and *Borrelia* spp. were also detected [28,29,30] and the PCR primers and cycling conditions are shown in Table 3. The DNAs extracted from the domestic animals and ticks in Qinghai infected with *A. ovis*, *A. bovis*, *A. phagocytophilum*, *Theileria ovis*, *Theileria sinensis*, and *Candidatus Rickettsia jingxinensis* were used as positive controls. The PCR products were detected by 1% agarose gel electrophoresis with M5 Hipure Next III Gelred (Mei5 Biotechnology Co., Ltd., Beijing, China) stained.

### 4.3. Sequencing and Phylogenetic Analysis

The PCR products from positive samples were sequenced at BGI Sequencing (Beijing, China) and subjected to BLAST searches for nucleotide sequence analysis and alignments. Phylogenetic trees were constructed using the neighbor-joining method executed with Kimura 2-parameter model in MEGA X. Bootstrap values were assessed with 1000 bootstrap replicates. The representative nucleotide sequences of this study have been deposited in the GenBank database under accession number MW147462 for *A. ovis*, MW142384 for *A. bovis*, MW142385 for *A. phagocytophilum*, and MW142379 for *T. ovis*.

### 4.4. Data Analysis

The data were grouped into three variables in terms of sheep keds gender and the region and the altitude of the sampling sites. Differences in infection rates of each group were statistically calculated using the Chi-square test in SPSS 25.0. A *p*-value of <0.05 was considered significant.

## Figures and Tables

**Table 1:** Detection of *Anaplasma* and *Theileria* pathogens in 92 ked pools at various geographic sites.

County/Average Altitude	Number of Pooled	Number of Infected (n)/Infection Rate (%)			
		*A. ovis*	*A. bovis*	*A. phagocytophilum*	*T. ovis*
Xunhua/3000 m	8	6/75.0	3/37.5	1/12.5	8/100
Maqin/3800 m	37	10/27.0	1/2.7	1/2.7	32/86.5
Dari/4100 m	15	4/26.7	2/13.3	0/0	14/93.3
Zhiduo/4100 m	32	16/50.0	10/31.3	7/21.9	28/87.5
Total	92	36/39.1	16/17.4	9/9.8	82/89.1

**Table 2:** Patterns of *Anaplasma* and *Theileria* pathogens positive rates in 92 *M. ovinus* pools, grouped by *M. ovinus* gender, the region, and altitude of the sampling sites.

Group		Number of Pooled	Number of Infected (n)/Infection Rate (%)							
			*A. ovis*	*p*-Value	*A. bovis*	*p*-Value	*A. phagocytophilum*	*p*-Value	*T. ovis*	*p*-Value
Region	Haidong	8	6/75.0	**0.01**	3/37.5	**0.003**	1/12.5	**0.011**	8/100	0.581
	Golog	52	14/26.9		3/5.8		1/1.9		46/88.5	
	Yushu	32	16/50.0		10/31.3		7/21.9		28/87.5	
Gender	Female	41	18/43.9	0.40	5/12.2	0.238	6/14.6	0.160	36/87.8	0.714
	Male	51	18/35.3		11/21.7		3/5.9		46/90.2	
Altitude	3000 m	8	6/75.0	**0.033**	3/37.5	**0.007**	1/12.5	0.169	8/100	0.537
	3800 m	37	10/27.0		1/2.7		1/2.7		32/86.5	
	4100 m	47	20/42.6		12/25.5		7/14.9		42/89.4	

Bold typeface indicates significant difference.

**Table 3:** Primers used for vector-borne pathogens detection in *M. ovinus*.

Pathogens	Target Gene	Methods	Primers		Product (bp)	Annealing T (°C)	Reference
*A. bovis*	16S rRNA	PCR	EE1 EE2	5′-TCCTGGCTCAGAACGAACGCTGGCGGC-3′ 5′-AGTCACTGACCCAACCTTAAATGGCTG-3′	1430	55	[27]
		nPCR ^†^	AB1f AB1r	5′-CTCGTAGCTTGCTATGAGAAC-3′ 5′-TCTCCCGGACTCCAGTCTG-3′	551	60	[27]
*A. phagocytephilum*	16S rRNA	PCR	EE1 EE2	5′-TCCTGGCTCAGAACGAACGCTGGCGGC-3′ 5′-AGTCACTGACCCAACCTTAAATGGCTG-3′	1430	55	[27]
		nPCR	SP2f SP2r	5′-GCTGAATGTGGGGATAATTTAT-3′ 5′-ATGGCTGCTTCCTTTCGGTTA-3′	641	60	[27]
*A. centrale*	16S rRNA	PCR	EE1 EE2	5′-TCCTGGCTCAGAACGAACGCTGGCGGC-3′ 5′-AGTCACTGACCCAACCTTAAATGGCTG-3′	1430	55	[27]
		nPCR	AC1f AC1r	5′-CTGCTTTTAATACTGCAGGACTA-3′ 5′-ATGCAGCACCTGTGTGAGGT-3′	426	60	[27]
*A. platys*	16S rRNA	PCR	EE1 EE2	5′-TCCTGGCTCAGAACGAACGCTGGCGGC-3′ 5′-AGTCACTGACCCAACCTTAAATGGCTG-3′	1430	55	[27]
		nPCR	APf APr	5′-AAGTCGAACGGATTTTTGTC-3′ 5′-CTTTAACTTACCGAACC-3′	506	60	[27]
*A. ovis*	msp4	PCR	oMSP4Fw oMSP4Rev	5′-TGAAGGGAGCGGGGTCATGGG-3′ 5′-GAGTAATTGCAGCCAGGGACTCT-3′	347	62	[26]
*A. marginale*	msp4	PCR	mMSP4Fw mMSP4Rev	5′-CTGAAGGGGGAGTAATGGG-3′ 5′-GGTAATAGCTGCCAGAGATTCC-3′	344	60	[26]
*A. capra*	gltA	PCR	Outer-f Outer-r	5′-GCGATTTTAGAGTGYGGAGATTG-3′ 5′-TACAATACCGGAGTAAAAGTCAA-3′	1031	55	[25]
		nPCR	Inner-f Inner-r	5′-TCATCTCCTGTTGCACGGTGCCC-3′ 5′-CTCTGAATGAACATGCCCACCCT-3′	594	60	[25]
	16s rRNA	PCR	Capra-F Capra-R	5′-GCAAGTCGAACGGACCAAATCTGT-3′ 5′-CCACGATTACTAGCGATTCCGACTTC-3′	1261	60	[26]
Piroplasm	18S rRNA	PCR	Piro1-S Piro3-AS	5′-CTTGACGGTAGGGTATTGGC-3′ 5′-CCTTCCTTTAAGTGATAAGGTTCAC-3′	1410	55	[27]
		nPCR	PIRO-A1 PIRO-B	5′-CGCAAATTACCCAATCCTGACA-3′ 5′-TTAAATACGAATGCCCCCAAC-3′	430	55	[27]
BDV ^‡^	5′-UTR	RT-PCR	PBD1 PBD2	5′-TCGTGGTGAGATCCCTGAG-3′ 5′-GCAGAGATTTTTTATACTAGCCTATRC-3′	225	54	[14]
*Rickettsia* spp.	16S rRNA	PCR	Rick-16S-F3 Rick-16S-R4	5′-ATCAGTACGGAATAACTTTTA-3′ 5′-TGCCTCTTGCGTTAGCTCAC-3′	1284	58	[28]
	OmpA	PCR	Rr190.70 Rr190.701	5′-ATGGCGAATATTTCTCCAAAA-3′ 5′-GTTCCGTTAATGGCAGCATCT-3′	632	50	[30]
*Borrelia* spp.	ITS 5S-23S rRNA	PCR	Outer23SN1 Outer23SC1	5′-ACCATAGACTCTTATTACTTTGAC-3′ 5′-TAAGCTGACTAATACTAATTACCC-3′	380	52	[29]
		nPCR	Inter-23SN2 Inter-23SC2	5′-ACCATAGACTCTTATTACTTTGACCA-3′ 5′-GAGAGTAGGTTATTGCCAGGG-3′	230	55	[29]

^†^: nested PCR; ^‡^: Border disease virus.

## Data Availability

The data presented in this study are available on request from the corresponding author.
